# Contextual Determinants in Alcohol, Tobacco and Cannabis Consumption, Mood and Bullying during Adolescence

**DOI:** 10.3390/ijerph18168480

**Published:** 2021-08-11

**Authors:** Ainara Díaz-Geada, Núria Obradors-Rial, Antoni Baena, Ester Teixidó-Compañó, Ester Colillas-Malet, Narmeen Mallah, Lucía Moure-Rodríguez, Francisco Caamaño-Isorna, Tivy Barón-Garcia

**Affiliations:** 1 Department of Public Health, University of Santiago de Compostela, 15782 Santiago de Compostela, Spain; ainara.geada@usc.gal (A.D.-G.); narmeen.mallah@usc.es (N.M.); lucia.moure.rodriguez@usc.es (L.M.-R.); 2 Faculty of Health Sciences of Manresa, University of Vic—Central University of Catalonia (UVic-UCC), 08242 Manresa, Spain; nobradors@umanresa.cat (N.O.-R.); eteixido@umanresa.cat (E.T.-C.); ecolillas@umanresa.cat (E.C.-M.); sbaron@umanresa.cat (T.B.-G.); 3 Faculty of Health Sciences, Universitat Oberta de Catalunya (UOC), 08018 Barcelona, Spain; abaenag@uoc.edu; 4 Tobacco Control Unit, Cancer Control and Prevention Programme, Institut Català d’Oncologia—ICO, Av. Granvia de L’Hospitalet 199-203, 08908 Barcelona, Spain; 5 Biomedical Research Networking Center for Epidemiology and Public Health (CIBERESP), 28029 Madrid, Spain; 6 Health Research Institute of Santiago de Compostela (IDIS), 15706 Santiago de Compostela, Spain

**Keywords:** adolescents, social inequalities, territorial inequalities, socioeconomic factors, drug use, bullying, negative mood

## Abstract

The present study aimed to explore the differences in the consumption of alcohol, tobacco and cannabis, mood and bullying between adolescents. A cross-sectional study was carried out in five regions of Northern Spain (one in Galiza and four in central Catalonia) that share similar socioeconomic characteristics and encompass around 10,000 inhabitants each. Students living in Burela, Galiza (*N* = 71) were compared to those of Central Catalonia (*N* = 193). The independent variable was the municipality of residence. The dependent variables encompassed: weekly available pocket money, Family Affluence Scale, self-classified academic qualification, place of origin, alcohol consumption, tobacco and cannabis smoking, negative mood and bullying. The mean age and their 95% confidence intervals (95% CI) of participants were similar between the regions (Burela: 15.90 years (15.68–16.13) and Central Catalonia: 15.36 years (15.28–15.44)). More than half of the participants were females (Burela, Galiza (53.5%) and Catalonia (54.9%)). Prevalence ratios (PR) and their 95% CI were estimated using Poisson regression models. In comparison with adolescents from Burela (Galiza), those living in Central Catalonia had higher prevalence of diverse academic levels (adjusted PR = 3.92 (1.78–8.66)), tobacco consumption (adjusted PR = 2.41 (1.47–3.97)) and negative mood (adjusted PR = 5.97 (3.05–11.70)). Even when dealing with regions with similar socioeconomic characteristics and number of inhabitants, differences exist in terms of the socioeconomic level, tobacco consumption, mood and bullying, as reported by adolescents.

## 1. Introduction

Individual and contextual socioeconomic conditions affect the lifestyle and health status of individuals, leading to social inequalities in health [1,2,3]. Evaluating social inequalities in health with respect to socioeconomic context is complicated by variations in the conceptual frameworks, methodological heterogeneity [4,5] and differences in the interpretation guidelines of related indexes and indicators [6,7]. 

Some studies have investigated the social differences between rural and urban populations by exclusively using the number of inhabitants as an indicator [8,9,10]. Such a method of classification may limit the findings generated by those studies as it does not consider other properties specific to the studied populations [3].

Research on social inequalities in health has been mainly centred in large populations (>10,000 inhabitants), while only a few studies have reported poorer access to social services, health care facilities or medically inappropriate behaviours in populations considered as rural (<10,000 inhabitants) in comparison with other larger populations [11,12]. Relying on population size exclusively in studying the social inequalities in health would lead to generalizing findings of numerous indicators that could differ between settings, even in regions with a similar number of inhabitants. 

The characteristics of the environment where people live, as one of the potential determinants in health, could affect the health behaviors of the population. For instance, studies carried out in Spain have suggested that behaviors such as eating habits differ between populations living in north and east Spain, pointing out, therefore, the need for surveillance of different risk factors for health in these populations [13]. The prevalence of other risky behaviors such as substance consumption also differs between regions in Spain [14]. These variations could be related to the different socioeconomic status between regions [15] and might affect the physical [16] and mental health status [17]. Accordingly, we hypothesize that these populations, despite sharing certain socioeconomic characteristics and belonging to the same country and public health system, could present differences in factors that may influence health behaviors. Evaluating those factors could allow a better management of social and health services and resources.

Extrapolating the findings of contextual socioeconomic conditions (income or size of a municipality) that are routinely used to explain the health status or health behaviour of another population that shares similar characteristics to the population under study, may introduce important biases. This is particularly relevant in small municipalities [18]. In addition to the contextual socioeconomic factors, other indicators such as family income, access to social and health services, public transport, leisure options [8,19], or demographic density may play an important role in explaining the health status or health behaviours of adolescents [20,21]. Evaluating the association of population characteristics other than the number of inhabitants will help prevent misclassifying a population. The misclassification of populations can lead to erroneously associating a determinant or a characteristic with a specific outcome in the population. To study the health determinants and improve the health level of a population, it is crucial to adequately allocate it in the correct socioeconomic and sociodemographic categories, otherwise, health behaviors and health outcomes in different populations will be erroneously generalized, and fundamental determinants for the design and success of health interventions will be ignored. Accordingly, healthcare professionals must consider the contextual determinants of health that are specific to each population in order to design population-specific preventive strategies.

In Europe, including Spain, most of the research on the association of socioeconomic context with health status and behaviour has focused on comparing mortality and comorbidity rates between large municipalities [2,6,15,20,21,22]. Some other studies have reported differences between rural and urban populations [8,9,10,23,24,25]. However, to-date, none have compared the health status and health behaviours between small municipalities while taking into account the factors related to inequalities in health, such as ethnicity, gender and social class.

Health behaviours acquired in adolescence can have implications on health status in adulthood. Mental health problems during adolescence lead to increased morbidity and mortality. Suffering from bullying during adolescence affects physical and mental health [26]. Moreover, risky behaviors such as the initiation of substance consumption, e.g., cannabis dependence in youth, are related to great psychological distress [27]. In the Galician population, which forms a part of this study, an association was found between the following variables: negative mood, alcohol, tobacco and cannabis consumption, and having suffered bullying. Therefore, it is relevant to check if these associations are maintained across populations with similar socioeconomic characteristics.

For these reasons, in the present study, we investigated determinants, other than population size, of social inequalities in health that might influence health behaviour with a sociological approach. We explored differences in alcohol, tobacco and cannabis consumption, negative mood and bullying in adolescents from five small regions in the northwest and northeast of Spain (Galiza and Catalonia), which share similar socioeconomic properties including population size. 

### Setting

This study was carried out in Spain, Southern Europe. Spain is divided into territories known as autonomous communities that differ in their socioeconomic characteristics and health indicators.

Participants in this study were recruited from five municipalities belonging to two Spanish autonomous communities with similar socioeconomic characteristics: Burela in the northwest (Galiza) and Centelles, Torelló, Sant Joan de Vilatorrada and Sant Fruitós de Bages in the northeast (Catalonia). Each of the five municipalities has a population size of approximately 10,000 inhabitants.

To select the most similar municipalities, the following indicators were used: personal income tax, number of inhabitants, percentage of population of non-Spanish origin and the distance to the region capital.

Burela differs from the other four municipalities by being a multicultural town that harbours more than fifty different nationalities within its 9500 inhabitants. The multicultural coexistence in Burela raises new challenges in the search for better social integration in Galiza, a context with a short history of immigrants’ reception. Studies have pointed out the various effects of socioeconomic context and ethnicity, as well as the influence of acculturation process on adolescents [28]. As the population of adolescents is very diverse and young, it will be interesting to study the potentially existing inequalities in such a paradigmatic context. 

Burela is a very different population compared to most of the Galician municipalities with respect to its number of inhabitants. Therefore, we compared Burela to four municipalities, Centelles, Torelló, Sant Joan de Vilatorrada and Sant Fruitós de Bages, that were selected based on their socioeconomic and demographic characteristics and which are more similar to Burela than the rest of municipalities included in the study of Obradors and colleagues [10]. In Spain, the access to health care is universal and free. Burela has one reference public hospital (Hospital Público da Mariña, Burela) and one center of primary care services. Likewise, each of the four Catalan municipalities have one reference hospital (Hospital General de Vic in Centelles and Torelló; Hospital Sant Joan de Déu, in Manresa for the municipalities Sant Joan de Vilatorrada and Sant Fruitós de Bages) and one center of primary care services.

## 2. Materials and Methods

### 2.1. Study Design and Population

A cross-sectional study was carried out to compare the socioeconomic characteristics and health behaviours of individuals in middle-stage adolescence. The participants were second year high school students (15 years old) recruited from five municipalities in Northern Spain. To select the most similar municipalities, the following indicators were used: personal income tax, number of inhabitants, percentage of population of non-Spanish origin and the distance to the region’s capital.

Students were recruited in two waves, 2012 and 2015. Burela (Galiza) students were recruited from two high schools in 2015, while those in Centelles, Torelló, Sant Joan de Vilatorrada and Sant Fruitós de Bages (Catalonia) were recruited in a previous study in 2012 [10]. In Burela, taking into account the limited size of this population and its high accessibility, we adopted an exhaustive sampling strategy by targeting the entire population. In Catalan municipalities, we followed cluster sampling with the class used as the sampling unit. We stratified the schools by their type (private/subsidized or public), township size (fewer than 2000 inhabitants classified as rural, 2001–10,000 inhabitants classified as intermediate and more than 10,000 inhabitants classified as urban) [29] and socioeconomic position of the school township (average income tax for the town). High schools that refused to participate in the study were replaced by another high school with the same characteristics based on the strata until we reached the target sample size needed to be representative of the Catalan region according to the variables used for the stratification. We then, performed a random sampling to select the classrooms

### 2.2. Data Collection

To collect the data we used a modified version of the questionnaire FRESC (Factors de Risc en Estudiants de Secundària). FRESC was designed by the Public Health Agency of Barcelona in order to explore the emerging risk behaviours in adolescents [30]. FRESC has demonstrated good metric qualities in several studies carried out in the Spanish adolescent population [31,32]; hence, the instrument is reliable to be used in our study. The same questionnaire was used to measure the target variables in the entire sample.

The questionnaire was anonymous and self-administered. The confidentiality of the data was guaranteed throughout the study.

### 2.3. Definition of Variables

#### 2.3.1. Independent Variable

The main independent variable was the municipality of residence of the students. Each of the four Catalonian municipalities (Centelles, Torelló, Sant Joan de Vilatorrada and Sant Fruitós de Bages) were individually compared with Burela municipality at first, and then compared as a group (Burela vs. Catalonia).

#### 2.3.2. Dependent Variables

We assessed outcomes related to lifetime substance use, including lifetime experimental consumption of alcohol, tobacco and cannabis. The three variables were binary (yes vs. no). We measured the perception of risk from cannabis smoking by estimating the proportion of adolescents who considered cannabis very dangerous to the total number of students.

Bullying is a composite variable generated from the following three items: In the past twelve months: Has someone ever laughed at you or insulted you at school or on the way to school?; Have you ever been beaten, attacked, or threatened at school or on the way to school?; Do your colleagues sometimes marginalize you? The students were requested to answer each of these questions by selecting from the following five choices: never, once, twice, three times or more than three times. Adolescents were considered to have suffered bullying if they responded to any of those three questions by “three times or more” or if they chose “at least once” to answer each of those three items [33].

We measured the mental health status of adolescents by assessing the “negative mood” of the students. Students were asked: How many times have you felt: very tired doing normal activities? You had difficulty sleeping or staying asleep? Out of place, sad, or depressed? Hopeless facing the future? Nervous or tense? Bored with things? Threatened by another student at school? Students answered on the mood questions using a 0 to 4 Likert scale where 0 and 4 meant never and always, respectively. The mood variable was then transformed into binary variable: “Never, Almost never or Sometimes” versus “Frequently or Always”. People who had answered “Always” or “Frequently” on at least three of the mood items were classified as having negative mood [32].

#### 2.3.3. Other Included Variables

Weekly pocket money: students were classified into two groups: having ≤ EUR 10 or having > EUR 10 for their personal weekly expenses.

Family Affluence Scale (FAS): Family Affluence Scale is a widely used scale to measure the socioeconomic status in the adolescent population [34]. FAS has been validated in European populations [35] and widely used in populations similar to ours [9,10,23,31]. Students were asked whether their family have a transport means (car or van), whether they have their own room, the number of computers they have and how many times they have been on a holiday with their family in the previous year. Answers on these questions were classified into: low FAS (considered as having disadvantaged socioeconomic status) for a score of 0–3 points; average FAS (average socioeconomic status) if the score was 4–5 points; and high FAS (socioeconomically advantaged) with 6–7 points. 

In addition, we inquired about self-classification referred gender and to academic qualifications through the question: In reference to your classmates, in which academic level would you classify yourself? Answers on academic qualifications were converted into two categories: low versus medium–high. We also classified the students according to their place of origin: “native” (the adolescent and one or both of his/her parents were born in Spain) and “immigrant” (the adolescent and/or both parents were born outside Spain).

### 2.4. Statistical Analysis

A descriptive statistical analysis was performed for the overall study sample as well as for Galician and Catalonian adolescents, separately.

The prevalence ratio of the different variables was calculated for Burela and for the four Catalonian municipalities, individually and as a group. To analyse the association between variables in both populations (Galiza and Catalonia), univariate and multivariate Poisson regression models were applied to estimate the prevalence ratios (PR) with their respective 95% confidence intervals (95% CI). PRs were defined as the prevalence of the outcome in the exposed population over that in the non-exposed population [36]. We estimated the PRs of the different outcome for Burela as well as for the four Catalonian municipalities, individually and as a group (Catalonia).

The analyses were carried out using STATA 15.0 statistical package. 

## 3. Results

The final sample size encompassed 264 students (71 from Burela, 21 from Centelles, 29 from Torelló, 100 from Sant Joan de Vilatorrada and 43 from Sant Fruitós de Bages). Table 1 represents the sociodemographic characteristics of the study population. More than half of the students in Burela, Galiza (53.5%) and Catalonia (54.9%) were females. The proportion of immigrants in Burela was higher than that in Catalonia (23.5% vs. 13.5%), although the difference was not statistically significant (*p* = 0.054). 

### 3.1. Socioeconomic Differences

Regarding the socioeconomic level, both populations (Galiza and Catalonia) were similar in terms of weekly pocket money (Table 1). Catalan students showed a higher FAS score (62.7%) than those of Burela (45.7%). 

All students reported more than 80.0% prevalence of alcohol consumption. The prevalence of ever having smoked tobacco or cannabis was higher in Catalan students (61.6% and 35.8%, respectively) than in Galician students (18.8% and 8.5%, respectively).

The prevalence of medium-to-low FAS in adolescents from Sant Joan de Vilatorrada (PR = 0.70 (95% CI: 0.50–0.97)) and (adjusted PR = 0.63 (95% CI: 0.41–0.97)) and Sant Fruitós de Bages (PR = 0.68 (IC95%: 0.44–1.07)) and (adjusted PR = 0.59 (IC95%: 0.59–0.99)) was higher than that in adolescents from Burela (Table 2 and Table 3). 

This effect was maintained in the analysis of the four Catalan municipalities together (adjusted PR = 0.64 (IC95%: 1.47–3.97)) (Table 4). 

The self-reported academic qualification was more heterogenous among Catalan than Galician students. A higher probability that the students classified themselves in a lower academic level than their colleagues was observed in the comparison of each of the four Catalonian municipalities to Burela, as it was indicated by the crude PR (Table 2) as well as by the multivariant analysis adjusted by municipalities: Centelles ([adjusted PR = 3.15 (IC95%: 1.12–8.87)), Torelló (adjusted PR = 3.15 (IC95%: 1.17–8.04)), Sant Joan de Vilatorrada (adjusted PR = 4.32 (IC95%: 1.93–9.69)) and Sant Fruitós de Bages (adjusted PR = 3.96 (IC95%: 1.64–9.55)) (Table 3) as well as in the pooled analysis of the four municipalities (adjusted PR = 3.93 (95% CI: 1.78–8.66)) (Table 4).

### 3.2. Differences in Alcohol, Tobacco and Cannabis Consumption

As for the consumption of alcohol, tobacco and cannabis, the prevalence of reporting having ever smoked tobacco by Catalan students was twice that found for students from Burela. This finding was obtained in the individual analysis: Centelles (adjusted PR = 2.66 (IC95%: 1.56–4.53)), Torelló (adjusted PR = 2.24 (IC95%: 1.31–3.81)), Sant Joan de Vilatorrada (adjusted PR = 2.36 (IC95%: (1.43–3.91)) and Sant Fruitós de Bages (adjusted PR = 2.58 (IC95%: (1.53–4.34)) (Table 3) as well as in the grouped analysis (adjusted PR = 2.41 (95%CI: 1.47–3.97)) (Table 4). 

The perception of risk from cannabis smoking was rated higher by Catalonian students (adjusted PR = 0.76 (95%CI: 0.57–0.95)) than by Galician students (Table 4). In the separate analysis of each Catalonian municipality, the higher prevalence of the perception of risk of cannabis was only observed for students from Sant Fruitós de Bages (adjusted PR = 0.61 (95% CI: 0.41–0.91)) in the separate multivariant analysis of the Catalonian municipalities (Table 3).

### 3.3. Differences in Mood and Bullying

The prevalence of negative mood in Catalonian students was substantially higher than that in Galician students, with the highest PR observed in Sant Fruitós de Bages (adjusted PR = 6.44 (95% CI: 3.24–12.63)) (Table 3). 

The prevalence of suffering from bullying in Catalonia was considerably lower than that in Burela, Galiza, as it was revealed by the individual analysis of the Catalonian municipalities: Torelló (adjusted PR = 0.05 (IC95%: (0.01–0.38)), Sant Joan de Vilatorrada (adjusted PR = 0.07 (IC95%: (0.02–0.22)) and Sant Fruitós de Bages (adjusted PR = 0.07 (IC95%: (0.02–0.30)) (Table 3) and by the grouped analysis (adjusted PR = 0.08 (95% CI: 0.03–0.25)) (Table 4). 

## 4. Discussion

The findings of the present study suggest that Galician (Burela) students differ in their health status and health behaviours from Catalonian students, despite the similarity in the socioeconomic conditions and the number of inhabitants in the compared municipalities. Nevertheless, the four Catalonian populations showed similarity in their health status and health behaviours.

The most notable differences were the following: (1) The self-classified academic qualification was more diverse among the Catalan students than the Galician students; (2) Experimental tobacco consumption was more frequent in the Catalan student population; (3) Negative mood was reported to a greater extent by Catalan students; (4) Bullying was more likely to take place among Galician (Burela) students.

Our findings about the higher prevalence of tobacco smoking and negative mood among Catalan adolescents compared to their Galician counterparts, corroborates the relationship between tobacco smoking and low mood. In fact, previous studies have suggested the presence of an association between tobacco and mood [37], however, they have not established the direction of that association. When each of the four Catalonian municipalities were analyzed separately, we observed a higher prevalence of cannabis use by Catalan than Galician students despite most of the Catalan students showing an awareness of the risk associated with cannabis smoking. On the one hand, this observation could be provoked by other determinants with more influence on the decision to try cannabis such as social network (peers and family) and social norms. Besides, Catalonian adolescents showed a lower perception of their academic qualification than Galician students, which could be due to a worse school self-esteem for the former than the latter [31,37]. This could explain why no differences appeared in cannabis consumption in the multivariate analyses. On the other side, a higher socioeconomic level is known to contribute to increased substance consumption [31]. In our study, although the Catalonian and Galician (Burela) municipalities have similar personal income tax (PIT), Catalonian adolescents had a higher FAS, probably due to scale characteristics and cultural reasons and, thus, this could have induced the differences in cannabis consumption. Nonetheless, the statistical significance of the association between cannabis smoking and place of residence was lost in the grouped analysis of Catalonian municipalities, probably due to the limited sample size.

Our findings on the prevalence of cannabis consumption per municipality are in line with those of previous studies, which reported a lower prevalence in Galiza than in Catalonia [38]. Our data also showed that the prevalence of any substance consumption, including cannabis and alcohol, in each of the four Catalonian municipalities (Centelles, Torelló, Sant Joan de Vilatorrada, Sant Fruitós de Bages) was higher than the average prevalence reported for their autonomous community (Catalonia). In addition, the prevalence of substance consumption in Burela is lower than Galiza, expect for alcohol consumption. This could indicate that there are important differences between socioeconomically similar municipalities that we must understand.

In our study, Catalonian students had lower prevalence of bullying but higher prevalence of negative mood than Galician students. This contradicts findings from previous studies carried out with Galician students, which concluded that negative mood is associated with bullying [31]. This difference could stem from the multicultural characteristics of this population, where we hypothesize that students from Burela who participated in our study differ culturally from the participants of earlier studies. Therefore, future studies are needed to clarify the relationship between bullying and mood.

The observed differences between the Galician municipality, Burela, and the four Catalonian municipalities could be due to the influence of specific health policies in each of the two autonomous communities focused on the target behaviours. Differences in health plans between Galiza and Catalonia have already been demonstrated [39]. In addition, legislative changes with respect to children, during the period of the economic crisis in Spain in 2008, caused a reduction in the resources in the municipalities and, consequently, magnified inequalities between regions in Spain, especially in small municipalities [40].

The local administration could benefit from their proximity to citizens to play a key role in enhancing the knowledge of their population and facilitating intervention programs [41]. This would permit the reduction of inequalities and improve life conditions [40,42].

Our study suffers from three main limitations: (1) The comparison of a single Galician municipality such as Burela, gives evidence of the need to study more Galician populations with similar socioeconomic characteristics to confirm our hypothesis. (2) Given the cross-sectional design of the study, the validity of our conclusions could be limited by the difficulty of differentiating between the cause and the effect. However, in our study, the stability of the outcomes, specifically those related to experimenting with alcohol consumption and tobacco and cannabis smoking, permitted the obtaining of similar results to those of a longitudinal design. (3) Comparing data from populations studied in 2012 with those from 2015 may have introduced some bias because of possible temporal differences. However, in the period that elapsed between the data collection, no large divergences occurred in some of the studied behaviours in these territories [38]. (4) There could be a possible disparity bias because of the comparison of Catalonian municipalities with 193 respondents (21 from Centelles, 29 from Torelló, 100 from Sant Joan de Vilatorrada and 43 from Sant Fruitós de Bages) and a single Galician municipality of Burela, which could seem an unbalanced sample. However, we want to explore the cultural and contextual influences in territories that, even though they are disparate, share economic and demographic indicators.

As for the strengths of this study: (1) By being based on small populations, it allowed us to measure a reality that is rarely the focus of research in our field, despite the important fraction of the population represented by these municipalities and the reported differences regarding alcohol consumption and its consequences [10]; (2) Avoiding the dichotomous classification of rural–urban, and opting to choose similar populations, allowed us to explore the potential differences in health and to overcome the limitations of generating health policies according to the number of inhabitants, exclusively; (3) The individual factors have traditionally been the most studied in terms of their influence on health behaviour and its consequences on adolescents [43]. Therefore, the present study is the first to compare health behaviours in adolescents from two unrelated territories in Spain, showing a new perspective of the studies in these populations.

## 5. Conclusions

There are differences in the self-classified academic level, tobacco smoking, negative mood and bullying between adolescents in Galician and Catalonian municipalities that have similar socioeconomic characteristics and number of inhabitants. Our findings reflect the need for further research with different scales and methodologies in small populations, in which characteristics have been barely investigated in this field. These issues must be considered to improve the prioritization of measures and the effectiveness of preventive interventions that are developed in these populations. Local governments play a fundamental role in reducing health inequalities.

This study intends to provide a tool that investigates differences between small populations, which, despite being similar in terms of socioeconomic characteristics and number of inhabitants, have different health behaviours. This justifies the need to expand research in these populations, in which it is possible that very different health results are being generalized.

## Figures and Tables

**Table 1:** Characteristics of the populations of Central Catalonia and Burela (Galiza) (2012–2015).

	Central Catalonia		Burela		
	*N = 193*			*N = 71*	
	*N*	%	*N*	%	*p*-Value
Gender					
Female	106	54.9	38	53.5	0.839
Male	87	45.1	33	46.5	
Self-reported academic level					
High	68	35.2	19	27.1	<0.0001
Medium	102	52.9	44	62.9	
Low	23	11.9	7	0.1	
Place of origin					
Native	166	86.5	52	76.5	0.054
Immigrants	26	13.5	16	23.5	
Weekly pocket money					
≤EUR 10	109	56.5	34	50.8	0.417
>EUR 10	84	43.5	33	49.2	
Socioeconomic level (FAS)					
Low	12	6.2	12	17.2	0.007
Medium	60	31.1	26	37.1	
High	121	62.7	32	45.7	
Ever alcohol consumption					
Yes	168	87.1	57	80.3	0.170
No	25	12.9	14	19.7	
Ever tobacco smoking					
Yes	119	61.6	13	18.8	<0.0001
No	74	38.4	56	81.2	
Ever cannabis smoking					
Yes	69	35.8	6	8.5	<0.0001
No	124	64.2	65	91.5	
Risk perception of cannabis					
Moderate or not dangerous at all	94	48.7	55	77.5	<0.0001
Very dangerous	99	51.3	16	22.5	
Ever bullied					
Yes	10	10.7	9	12.7	0.037
No	183	89.3	62	87.3	
Negative mood					
Yes	132	68.4	8	11.3	<0.0001
No	61	31.6	63	88.7	

**Table 2:** Prevalence ratio (PR) and 95% confidence interval (CI) of socioeconomic characteristics, alcohol, tobacco and cannabis consumption, negative mood and bullying in adolescents from Catalonian municipalities compared to adolescents from Burela.

	Centelles		Torelló		Sant Joan de Vilatorrada		Sant Fruitós de Bages	
	PR	95% CI	PR	95% CI	PR	95% CI	PR	95% CI
Sex								
Male vs. Female	1.23	(0.8–1.92)	0.96	(0.60–1.55)	0.97	(0.69–1.35)	0.85	(0.54–1.33)
Self-classified academic qualification								
Low vs. Medium–High	2.86	(1.07–7.59)	2.76	(1.10–6.92)	3.90	(1.85–8.22)	3.49	(1.54–7.87)
Place of origin								
Immigrants vs. Native	0.20	(0.03–1.44)	0.58	(0.21–1.60)	0.64	(0.34–1.20)	0.61	(0.26–1.43)
Weekly pocket money								
≤ EUR 10 vs. > EUR 10	1.16	(0.74–1.81)	0.98	(0.62–1.53)	0.89	(0.64–1.24)	0.66	(0.40–0.63)
Socioeconomic level (FAS)								
Low–Medium vs. High	0.61	(0.32–1.17)	0.70	(0.42–1.17)	0.70	(0.50–0.97)	0.68	(0.44–1.07)
Ever alcohol consumption								
Yes vs. No	1.24	(1.11–1.40)	1.16	(0.99–1.35)	1.06	(0.92–1.22)	1.01	(0.84–1.22)
Ever tobacco smoking								
Yes vs. No	3.79	(2.16–6.64)	3.29	(1.87–5.81)	3.18	(1.90–5.34)	3.21	(1.86–5.55)
Ever cannabis smoking								
Yes vs. No	3.94	(1.48–10.48)	4.9	(2.03–11.82)	4.38	(1.95–9.83)	3.58	(1.47–8.73)
Risk perception of cannabis								
Moderate or not dangerous at all vs. very dangerous	0.49	(0.28–0.86)	0.67	(0.46–0.97)	0.67	(0.53–0.84)	0.57	(0.40–0.82)
Ever bullied								
Yes vs. No	*		0.27	(0.04–2.06)	0.47	(0.18–1.27)	0.55	(0.16–1.93)
Negative mood								
Yes vs. No	5.49	(2.63–11.46)	6.42	(3.22–12.83)	5.94	(3.05–11.60)	6.40	(3.24–12.63)

Burela was used as a reference category. * The sample size was not sufficient to estimate PR of having ever bullied in this municipality.

**Table 3:** Adjusted prevalence ratios (PR) of socioeconomic variables, alcohol, tobacco and cannabis consumption, negative mood and bullying in adolescents from Centelles, Torelló, Sant Joan de Vilatorrada and Sant Fruitós de Bages, compared to adolescents from Burela.

	Centelles		Torelló		Sant Joan Vilatorrada		Sant Fruitós Bages	
	_Adjusted_ PR	95% CI	_Adjusted_ PR	95% CI	_Adjusted_ PR	95% CI	_Adjusted_ PR	95% CI
Sex								
Male vs. Female	1.10	(0.66–1.84)	0.86	(0.50–1.48)	0.84	(0.55–1.28)	0.72	(0.43–1.20)
Self-classified academic qualification								
Low vs. Medium-High	3.15	(1.12–8.87)	3.07	(1.17–8.04)	4.32	(1.93–9.69)	3.96	(1.64–9.55)
Place of origin								
Immigrants vs. Native	0.30	(0.04–2.16)	0.77	(0.25–2.40)	0.75	(0.33–1.69)	0.70	(0.23–2.80)
Weekly pocket money								
≤ EUR 10 vs. > EUR 10	0.87	(0.54–1.40)	0.68	(0.42–1.11)	0.66	(0.45–0.96)	0.49	(0.29–0.83)
Socioeconomic level (FAS)								
FAS low-medium vs. FAS high	0.56	(0.30–1.04)	0.64	(0.36–1.16)	0.63	(0.41–0.97)	0.59	(0.35–0.99)
Ever tobacco smoking								
Yes vs. No	2.66	(156–4.53)	2.24	(1.31–3.81)	2.36	(1.43–3.91)	2.58	(1.53–4.34)
Ever cannabis smoking								
Yes vs. No	1.26	(0.51–3.07)	1.75	(0.80–3.83)	1.64	(0.77–3.51)	1.35	(0.60–3.05)
Risk perception of cannabis								
Moderated or not dangerous at all vs. very dangerous	0.58	(0.33–1.02)	0.81	(0.54–1.19)	0.75	(0.57–1.01)	0.61	(0.41–0.91)
Ever bullied								
Yes vs. No	*		0.05	(0.01–0.38)	0.07	(0.02–0.22)	0.07	(0.02–0.30)
Negative mood								
Yes vs. No	5.27	(2.50–11.12)	6.24	(3.11–12.54)	5.96	(3.05–11.68)	6.44	(3.24–12.83)

* The sample size was not sufficient to estimate PR in this municipality.

**Table 4:** Prevalence ratio (PR) and adjusted prevalence ratio of socioeconomic variables, alcohol, tobacco and cannabis consumption, negative mood and bullying in adolescents from Central Catalonia municipalities grouped together, compared to adolescents from Burela (Galiza).

	Central Catalonia		Burela	Central Catalonia		Burela
	PR	95% CI	Reference Category	_Adjusted_PR	95% CI	Reference Category
Sex						
Male vs. Female	0.97	(0.72–1.30)	1	0.87	(0.59–1.30)	1
Self-classified academic qualification						
Low vs. Medium-High	3.52	(1.70–7.31)	1	3.93	(1.78–8.66)	1
Place of origin						
Immigrants vs. Native	0.57	(0.33–1.01)	1	0.78	(0.36–1.71)	1
Weekly money available						
<= EUR 10 vs. > EUR 10	0.88	(0.66–1.18)	1	0.65	(0.46–0.92)	1
Socioeconomic level (FAS)						
FAS low–medium vs. FAS high	0.69	(0.52–0.91)	1	0.64	(0.43–0.93)	1
Ever alcohol consumption						
Yes vs. No	1.08	(0.95–1.23)	1	0.90	(0.77–1.04)	1
Ever tobacco smoking						
Yes vs. No	3.27	(1.98–5.41)	1	2.41	(1.47–3.97)	1
Ever cannabis smoking						
Yes vs. No	4.23	(1.91–9.32)	1	1.49	(0.71–3.11)	1
Risk perception of cannabis						
Moderated or not dangerous at all vs. very dangerous	0.63	(0.52–0.76)	1	0.76	(0.57–0.95)	1
Ever bullied						
Yes vs. No	0.41	(0.17–0.96)	1	0.08	(0.03–0.25)	1
Negative mood						
Yes vs. No	6.07	(3.13–11.75)	1	5.97	(3.05–11.70)	1

## Data Availability

All relevant data are included within the paper. Participants were informed that (I) participation was voluntary and students could drop out at any time without any consequence, (II) confidentiality and anonymity were guaranteed, and (III) the data would be guarded carefully by our research team for the purpose of this scientific study only. Anonymized data will be accessible upon request from the corresponding author.

## References

[1] Borrell C., Marí-Dell’Olmo M., Palència L., Gotsens M., Burström B., Domínguez-Berjón M.F., Rodríguez-Sanz M., Dzurova D., Gandarillas A., Hoffmann R. (2014). Socioeconomic inequalities in mortality in 16 European cities. Scand. J. Public Health.

[2] Borrell C., Pons-Vigués M., Morrison J., Diez E. (2013). Factors and processes influencing health inequalities in urban areas. J. Epidemiol. Commun. Health.

[3] Cummins S., Curtis S., Diez-Roux A.V., Macintyre S. (2007). Understanding and representing ‘place’ in health research: A relational approach. Soc. Sci. Med..

[4] Curtis S., Rees Jones I. (1998). Is there a place for geography in the analysis of health inequality?. Sociol. Health Illn..

[5] Ocaña-Riola R., Sánchez-Cantalejo C. (2005). Rurality Index for Small Areas in Spain. Soc. Ind. Res..

[6] Dominguez-Berjon F., Borrell C., Rodriguez-Sanz M., Pastor V. (2005). The usefulness of area-based socioeconomic measures to monitor social inequalities in health in Southern Europe. Eur. J. Public Health.

[7] Domínguez-Berjón M., Borrell C., Benach J., Pasarin M. (2001). Measures of material deprivation in small area studies. Gac. Sanit..

[8] Font-Ribera L., Garcia-Continente X., Pérez A., Torres R., Sala N., Espelt A., Nebot N. (2013). Driving under the influence of alcohol or drugs among adolescents: The role of urban and rural environments. Accid. Anal. Prev..

[9] Obradors-Rial N., Ariza C., Continente X., Muntaner C. (2019). School and town factors associated with risky alcohol consumption among Catalan adolescents. Alcohol.

[10] Obradors-Rial N., Ariza C., Muntaner C. (2014). Risky alcohol consumption and associated factors in adolescents aged 15 to 16 years in Central Catalonia (Spain): Differences between rural and urban areas. Gac. Sanit..

[11] Lu N., Samuels M.E., Kletke P.R., Whitler E.T. (2010). Rural-Urban Differences in Health Insurance Coverage and Patterns Among Working-Age Adults in Kentucky. J. Rural Health.

[12] Janzen B., Karunanayake C., Pahwa P., Dyck R., Rennie D., Lawson J., Pickett W., Bryce R., Hagel L., Zhao G. (2015). Exploring Diversity in Socioeconomic Inequalities in Health Among Rural Dwelling Canadians: Socioeconomic Inequalities in Health. J. Rural Health.

[13] Alemán J.A., Rentero M.P.Z., Montoro-García S., Mulero J., Garrido A.P., Leal M., Guerrero L., Ramos E., Ruilope L.M. (2016). Adherence to the “Mediterranean Diet” in Spain and Its Relationship with Cardiovascular Risk (DIMERICA Study). Nutrients.

[14] Meneses C., Romo N., Uroz J., Gil E., Markez I., Giménez S., Vega A. (2009). Adolescencia, consumo de drogas y comportamientos de riesgo: Diferencias por sexo, etnicidad y áreas geográficas en España. Trastor Adict..

[15] Gotsens M., Marí-Dell’Olmo M., Martínez-Beneito M.Á., Pérez K., Pasarín M.I., Daponte A., Puigpinós-Riera R., Rodríguez-Sanz M., Audicana C., Nolasco A. (2011). Socio-economic inequalities in mortality due to injuries in small areas of ten cities in Spain (MEDEA Project). Accid. Anal. Prev..

[16] Gallo V., Mackenbach J.P., Ezzati M., Menvielle G., Kunst A.E., Rohrmann S., Kaaks R., Teucher B., Boeing H., Bergmann M.M. (2012). Social inequalities and mortality in Europe--results from a large multi-national cohort. PLoS ONE.

[17] Melis G., Gelormino E., Marra G., Ferracin E., Costa G. (2015). The Effects of the Urban Built Environment on Mental Health: A Cohort Study in a Large Northern Italian City. Int. J. Environ. Res. Public Health.

[18] Karriker-Jaffe K.J. (2011). Areas of disadvantage: A systematic review of effects of area-level socioeconomic status on substance use outcomes. Drug Alcohol Rev..

[19] Donath C., Grässel E., Baier D., Pfeiffer C., Karagülle D., Bleich S., Hillemacher T. (2011). Alcohol consumption and binge drinking in adolescents: Comparison of different migration backgrounds and rural vs. urban residence a representative study. BMC Public Health.

[20] Elliott P., Wartenberg D. (2004). Spatial Epidemiology: Current Approaches and Future Challenges. EHP.

[21] Villalonga-Olives E., Marí-Dell’Olmo M., Gotsens M., Ramos M., Ramon J., Cabeza E., Borrell C. (2013). Análisis de desigualdades en mortalidad en áreas pequeñas: Queda camino por recorrer. Gac. Sanit..

[22] Bosque-Prous M., Kuipers M.A.G., Espelt A., Richter M., Rimpelä A., Perelman J., Federico B., Brugal M.T., Lorant V., Kunst A.E. (2017). Adolescent alcohol use and parental and adolescent socioeconomic position in six European cities. BMC Public Health Diciembre de.

[23] Obradors-Rial N., Ariza C., Rajmil L., Muntaner C. (2008). Socioeconomic position and occupational social class and their association with risky alcohol consumption among adolescents. Int. J. Public Health.

[24] Koutra K., Papadovassilaki K., Kalpoutzaki P., Kargatzi M., Roumeliotaki T., Koukouli S. (2012). Adolescent drinking, academic achievement and leisure time use by secondary education students in a rural area of Crete. Health Soc. Care Community.

[25] Jiang X., Li D., Boyce W., Pickett W. (2008). Alcohol consumption and injury among Canadian adolescents: Variations by urban-rural geographic status. J. Rural Health.

[26] Nepon T., Pepler D.J., Craig W.M., Connolly J., Flett G.L. (2020). A Longitudinal Analysis of Peer Victimization, Self-Esteem, and Rejection Sensitivity in Mental Health and Substance Use Among Adolescents. Int. J. Ment. Health Addict..

[27] Dorard G., Berthoz S., Phan O., Corcos M., Bungener C. (2008). Affect dysregulation in cannabis abusers: A study in adolescents and young adults. Eur. Child Adolesc. Psychiatry.

[28] Wickrama K.A.S., Elder G.H., Todd Abraham W. (2007). Rurality and Ethnicity in Adolescent Physical Illness: Are Children of the Growing Rural Latino Population at Excess Health Risk?. J. Rural Health.

[29] Instituto Nacional de Estadística Censos de Población y Viviendas 2001. http://www.ine.es/censo/es/glosario.html.

[30] Pérez A., Garcia-Continente X. (2013). Grup Col·Laborador Enquesta FRESC 2012. Informe FRESC 2012: 25 Anys D’enquestes a Adolescents Escolaritzats de Barcelona. Barcelona: Agència de Salut Pública de Barcelona. https://bcnroc.ajuntament.barcelona.cat/jspui/bitstream/11703/86684/4/12703Informe%202012.pdf.

[31] Díaz-Geada A., Espelt A., Bosque-Prous M., Obradors-Rial N., Teixidó-Compañó E., Isorna F.C. (2019). Association between negative mood states, psychoactive substances consumption and bullying in school-aged adolescents. Adicciones.

[32] Ahonen E.Q., Nebot M., Giménez E. (2007). Negative mood states and related factors in a sample of adolescent secondary-school students in Barcelona (Spain). Gac. Sanit..

[33] Garcia-Continente X., Pérez-Giménez A., Espelt A., Nebot Adell M. (2013). Bullying among schoolchildren: Differences between victims and aggressors. Gac. Sanit..

[34] Currie C., Molcho M., Boyce W., Holstein B., Torsheim T., Richter M. (2008). Researching health inequalities in adolescents: The development of the Health Behaviour in School-Aged Children (HBSC) family affluence scale. Soc. Sci. Med..

[35] Hobza V., Hamrik Z., Bucksch J., De Clercq B. (2017). The Family Affluence Scale as an Indicator for Socioeconomic Status: Validation on Regional Income Differences in the Czech Republic. Int. J. Environ. Res. Public Health.

[36] Espelt A., Mari-Dell’Olmo M., Penelo E., Bosque-Prous M. (2016). Applied Prevalence Ratio estimation with different Regression models: An example from a cross-national study on substance use research. Adicciones.

[37] Gonzálvez M.T., Espada J.P., Orgilés M. (2015). Estado de ánimo y consumo de tabaco en una muestra de adolescentes españoles. Rev. Lat. Am. Psicol..

[38] Ministerio de Sanidad Servicios Sociales e Igualdad (2018). Plan Nacional Sobre Drogas. Encuesta Estatal sobre el Uso de Drogas en Enseñanzas Secundarias (ESTUDES). https://pnsd.sanidad.gob.es/profesionales/sistemasInformacion/sistemaInformacion/encuestas_ESTUDES.htm.

[39] Borrell C., Peiró R., Ramón N., Isabel Pasarín M., Colomer C., Zafra E., Álvarez-Dardet C. (2015). Desigualdades socioeconómicas y planes de salud en las comunidades autónomas del Estado español. Gac. Sanit..

[40] López Ruiz V., Segura Del Pozo J., Pires Gómez M.P., Malmusi D., Vergara Duarte M., Pérez Sanz E. (2018). Municipalism and community health: Transforming through local government. SESPAS Report 2018. Gac. Sanit..

[41] Llorca E., Amor M.T., Merino B., Márquez F.J., Gómez F., Ramírez R. (2010). Healthy cities: A reference strategy in local public health policies. Gac. Sanit..

[42] Chamorro C., Díaz-Echenique L., Oliván J. (2019). Los servicios locales de salud pública: Estudio descriptivo de los municipios de Catalunya en 2016. Rev. Esp. Salud. Pública.

[43] Teixidó-Compaño E., Sordo L., Bosque-Prous M., Puigcorbé S., Barrio G., Brugal M.T., Belza M.J.J., Espelt A. (2018). Individual and contextual factors related to binge drinking among adolescents in Spain: A multilevel approach. Adicciones.

